# Research priority 6: How can occupational therapy services be more inclusive of mental and physical health?

**DOI:** 10.1177/03080226231197307

**Published:** 2023-08-31

**Authors:** Cate Bennett, Anita Mottram

**Affiliations:** 1 Nottinghamshire County Council, Nottinghamshire, UK; 2 Kirklees Council, Huddersfield, UK

The RCOT research and development strategy (RCOT, 2019) was published with the intent to inform, guide and direct the development of research competencies, knowledge and participation in research across the occupational therapy profession. Royal College of Occupational Therapist (RCOT) worked in collaboration with the James Lind Alliance, supported by people with lived experience and practitioners to identify the 10 key research priorities for occupational therapists (RCOT, 2021). To achieve this required a process of consultation and prioritisation of themes to reduce the original list down to the final 10 priorities that were perceived, by the consultees, to be the ones that mattered most.

Of those chosen, priority number 6 queries ‘How can OT services be more inclusive of mental and physical health?’ The intention is this should be a definitive research question but rather be a starting point from which research questions can be generated.

The Care Act (2014) stipulates a general responsibility of practitioners to promote individual’s well-being and provides key occupation-focused components that resonate with occupational therapists working in any sector.

As a profession, occupational therapists are trained in physical and mental health conditions and multiple interventions. We have transferable knowledge and skills useful in a range of services and sectors. If we continue to depict our profession’s principle concern as being the enablement of self-care, productive, and leisure occupations, instead of clearly articulating the links between occupational engagement and well-being, and between well-being and human rights, our potential contribution to promoting the right to well-being through occupation will be lessened (Hammell, 2018).

A strengths-based approach is an effective way to support people to live their own version of a satisfying life (Dunn, 2017). Occupational therapists know that recovery of a meaningful life is more important than the recovery of physical functions (Hammell, 2018).

Occupational therapy is by definition a complex intervention (Skivington et al., 2021) with the primary goal to improve the lives of individuals, groups and communities by enabling them to participate in meaningful activities (World Federation of Occupational Therapists, 2012). When people are unable to engage in the occupations they need to, expect to do or want to do, they experience occupational injustice. By increasing participation in occupation, people achieve a sense of well-being and belonging in their communities. By promoting this, occupational therapists can improve people’s outcomes, prevent and delay the need for health and social care services, contributing to a more effective use of resources.

The RCOT research and development strategy set as a baseline, a vision that within a decade, engaging in research would become a key part of every occupational therapist’s role, with the intent that this would help to demonstrate the quality and effectiveness of our interventions. It reinforced the UK policy framework for health and social care research (Health Research Authority, 2020) which documented that research was essential for promotion of effective health and well-being.

People with long-term physical health conditions are two to three times more likely to experience mental health problems, with depression and anxiety disorders being particularly common (Naylor et al., 2012).

*An example: where a person is having to live in one room because they cannot access their essential facilities in their home or wider community. Occupational therapists might not only focus on the psychological aspects of how the person engages in activities, motivating them to practice a skill, reassuring them, building their confidence until they can master the activity that matters to them, but also change the environment to remove barriers or introduce compensatory equipment.*

Occupation and well-being are complex phenomena interacting with different aspects of the physical and sociocultural realms (Pentland, 2021). The psychological impact of occupational imbalance, alienation or deprivation can be significant to the individual, both in the community and institutional settings, creating occupational and social injustice if people are unable to participate in the wider economic environment, meaning they, their family and carers often require support or interventions from health and social care services (Townsend and Wilcock, 2004). Integrated approaches that help a person look after both their physical and mental health offer an important opportunity to increase choice and control and improve the effectiveness of self-management (Naylor et al., 2016).

This research priority is woven through our professional identity, because irrespective of the occupational therapy service or sector, how can we delineate between the impact of physical and mental health when we are undertaking strength based, person centred, holistic approaches to enable people to live their lives in ways that matter to them?

Where occupational therapists become overly focused on the interventions they deliver, they miss the wider impact occupational therapy has on an individual’s goals, aspirations and well-being.

*For example, an elderly lady requiring a level access shower unfortunately died before the disabled facilities grant process was completed, although plans had been drawn up and builder’s contractors had identified a start date. The occupational therapist visited the widowed husband to check whether he would benefit from the adaptation. They documented his physical capabilities to access a shower with no recorded recognition* *of his changed circumstances had just changed, or of his ability to manage with day-to-day activities. How he was feeling emotionally, would he benefit from some support with managing grief? Should it be someone else’s job to be compassionate and understand the impact of his loss on his ability to engage in the occupations important to him?*

The case for integrated care for mental and physical health is both ethical and economic. By working more holistically, occupational therapists could offer positive outcomes for people, and greater value for money for integrated care systems (Naylor et al., 2016).

The 21st-century challenges for integrated care systems are high rates of mental health conditions among people with long-term physical health problems; poor management of ‘medically unexplained symptoms’ without an identifiable organic cause; reduced life expectancy among people with the most severe forms of mental illness largely attributable to poor physical health and limited support for the wider psychological aspects of physical health and illness (Naylor et al., 2016).

The National Health Service 5-year forward view reinforces the need for greater integration of physical and mental health services with the interplay between specialist service pathways and generic services, an area that could be investigated through research. The following are some potential research opportunities:

Why does our service provision force us to work in condition specific silos – mental health, physical health?
Is an integrated holistic practice model a better way forward, or are there benefits in maintaining specialisms?
How can the culture and behaviours be changed across health and social care to embed more holistic practice, a greater recognition of well-being or a return to generic practice?
How can we standardise practice, or refresh standards of practice to contribute to this need for cultural change?

We challenge practitioners to address this research priority to increase the evidence base of occupational therapy and enhance the research capacity of the profession by contributing to the development of the knowledge base to support practitioners in their roles and career progression and to the further evolution of occupational therapy. By doing this we can demonstrate our clinical and economic effectiveness, and through evidencing the impact of occupational therapy outcomes, we can make the profession indispensable to our integrated care systems and future health and social care developments.
